# Academic Response to Storm-Related Natural Disasters—Lessons Learned

**DOI:** 10.3390/ijerph15081768

**Published:** 2018-08-17

**Authors:** Jerris R. Hedges, Karam F. A. Soliman, Gene D’Amour, Dong Liang, Carlos E. Rodríguez-Díaz, Kenira Thompson, Josefina Romaguera, Silvia E. Rabionet Sabater, Richard Yanagihara

**Affiliations:** 1 John A. Burns School of Medicine, University of Hawaii-Manoa, Honolulu, HI 96813, USA; ryanagih@hawaii.edu; 2 College of Pharmacy & Pharmaceutical Sciences, Florida A&M University, Tallahassee, FL 32307, USA; karam.soliman@famu.edu; 3 Office of the President, Xavier University of Louisiana, New Orleans, LA 70125, USA; gdamour@xula.edu; 4 College of Pharmacy and Health Sciences, Texas Southern University, Houston, TX 77004, USA; liang_dx@tsu.edu; 5 Graduate School of Public Health, University of Puerto Rico-Medical Sciences Campus, San Juan, PR 00936, USA; carlos.rodriguez64@upr.edu; 6 Ponce Research Institute, Ponce Health Sciences University, Ponce, PR 00732, USA; kthompson@psm.edu; 7 Department of Obstetrics and Gynecology, School of Medicine, University of Puerto Rico-Medical Sciences Campus, San Juan, PR 00936, USA; josefina.romaguera@upr.edu; 8 College of Pharmacy, Nova Southeastern University, Fort-Lauderdale-Davie, FL 33314, USA; rabionet@nova.edu

**Keywords:** natural disaster, hurricane aftermath, academic institution resilience, health disparities, storm-related illness

## Abstract

On 30 October 2017, selected faculty and administrators from Research Centers in Minority Institutions (RCMI) grantee institutions gathered to share first-hand accounts of the devastating impact of Hurricanes Harvey, Irma, and Maria, which had interrupted academic activities, including research, education, and training in Puerto Rico, Florida, and Texas. The presenters reviewed emergency response measures taken by their institutions to maintain community health care access and delivery, the storm-related impact on clinical and research infrastructure, and strategies to retain locally grown clinical expertise and translational science research talent in the aftermath of natural disasters. A longer-term perspective was provided through a comparative review of lessons learned by one New Orleans-based institution (now more than a decade post-storm) in the aftermath of Hurricane Katrina. Caring for the internal and external communities associated with each institution and addressing the health disparities exacerbated by storm-related events is one key strategy that will pay long-term dividends in the survival of the academic institutions and the communities they serve.

## 1. Introduction

There are limited recent reports that describe the recovery of academic institutions from storm-related natural disasters, which impact the lives of institutional personnel and their families, compromise associated physical infrastructure, and impair academic operations. The ability of these institutions and affiliated employees to fulfill service obligations to their local community, contribute to the local economy, and continue academic (research and educational) missions is a product of storm-related harm to the institution and surrounding community. Such harm can be mitigated by appropriate anticipation of potential storm-related damage and a coordinated response. Smaller academic institutions may be particularly vulnerable to such storms, given limited resources to mount a coordinated response. To share valuable lessons associated with institutional responses from recent devastating storms impacting Research Centers in Minority Institutions (RCMI) grantee institutions, an observational report was prepared.

## 2. Methods

On 30 October 2017, selected faculty and administrators from RCMI grantee institutions gathered in Washington DC to discuss their recent first-hand institutional accounts of the devastating impact of Hurricanes Harvey, Irma, and Maria, which had interrupted academic activities in Puerto Rico, Florida, and Texas. The presenters addressed emergency measures taken at their institutions to maintain community health care access and delivery, address clinical and research infrastructure challenges, and retain locally grown clinical expertise and translational science research talent in the aftermath of these storm-related disasters.

Following a brief historical overview of the three storms to set the environmental context, relevant institutional experiences and lessons learned are summarized. A longer-term perspective was provided by the review of lessons learned and perspectives gained at one New Orleans-based institution (now more than a decade post-storm) in the aftermath of Hurricane Katrina. These summaries and lessons learned are presented in the historical order of storm occurrence beginning with Hurricane Katrina. 

## 3. Overview of Major Hurricanes Significantly Disrupting Costal Academic Operations

On 29 August 2005, Hurricane Katrina struck the Gulf Coast of the United States (U.S.). It is believed to be the largest—and the third strongest—hurricane to make U.S. continental landfall. The major damage was due to storm surge and rainfall-associated levee breaching in New Orleans and surrounding counties with massive flooding [1,2,3]. Hundreds of thousands of people in Louisiana, Mississippi, and Alabama were displaced from their homes, with estimated economic damage of more than $100 billion. The death toll was more than 1800, with most victims being senior citizens who could not evacuate or survive the challenges of the flooding. Up to 80% of New Orleans was underwater, including parts of the Louisiana State Health Sciences Center, Tulane University, and Xavier University of Louisiana campuses as well as associated faculty, staff, and student housing.

On 25 August 2017, Hurricane Harvey made landfall in southwest Texas, causing rainfall-triggered flooding in the Houston metropolitan area on 28 August 2017 and an estimated $125 billion in damages [4]. Over four days, many areas received more than 60 inches of rain. The resulting floods impacted hundreds of thousands of homes and displaced more than 30,000 people. An estimated 107 lives were lost from Hurricane Harvey.

In early September 2017, Hurricane Irma became the strongest storm known to exist in the open Atlantic region. It was the first Category 5 hurricane of the 2017 Atlantic hurricane season and caused widespread damage in the northeastern Caribbean and the Florida Keys [5]. Limited damage to Puerto Rico occurred as Hurricane Irma largely skirted the north side of the island on 6–7 September 2017. Nonetheless, evacuation plans were implemented and routine operations curtailed during the storm. One million of the estimated 3.4 million residents of Puerto Rico were without electrical power. Two people died during the rainstorms ahead of the hurricane and two died during the hurricane. As Puerto Rico was recovering (60,000 residents were still without power) from storm damage associated with Hurricane Irma, a second hurricane threatened the island.

On 20 September 2017, Hurricane Maria, a Category 4 hurricane, made direct landfall on the south side of Puerto Rico, traveling northwest across the entire island as a 50-mile-wide tornado [6]. For weeks following this second hurricane, there was little power on the island; as of 16 October 2017, 86% of the island’s 1.57 million electricity customers still had no power. In many rural settings, there continued to be little water to drink, to bathe in, or to flush toilets.

Hurricane Maria took off-line 85% of the 1360 cell phone towers on the island, and nearly a month later only 43% had been repaired. Many communities were isolated from the outside world for weeks. The extent of the infrastructure damage remains to be determined. One estimate by Moody’s Analytics, a financial services firm, estimated the storm could cost Puerto Rico $45 billion to $95 billion [7], while the National Hurricane Center reported an estimate of $90 billion [8]. Most hospitals had to run on generators for weeks, and fuel was limited in some regions. By mid-October, the Federal Emergency Management Agency (FEMA) reported that 64 of 67 hospitals were open. An early report of the impact of Hurricane Maria was provided by Zorrilla [9]. The death count from Hurricane Maria is still being determined, with 64 official storm-related deaths in Puerto Rico. A recent report by Kishore et al. [10] using household survey data estimated a total of 4645 excess deaths (95% CI, 793 to 8498) from September 20 through 31 December 2017—equivalent to a 62% increase in the mortality rate compared with the same period in 2016. Traditional methodologies (which review each death and assign the cause of death to either a storm-related event or to a “natural cause”) by design yield much lower, storm-associated mortality rates. The traditional approach commonly does not address the contributions of stress, disruption of medical care, physical exertion, and other phenomena leading to early mortality precipitated by the storm event.

## 4. Institutional Response and Lessons Learned

### 4.1. Lessons from Hurricane Katrina—Gene D’Amour

Prolonged flooding and structural damage, a breakdown in communication, and massive displacement of the population complicated the recovery from Hurricane Katrina. The Xavier University of Louisiana worked with FEMA and local businesses to attain operational support and temporary housing (e.g., FEMA purchased trailers) for employees and students. When the immediate flooding threat had passed, faculty/staff/students assisted with neighborhood assessments and support of survivors (including the rescue of those who were isolated by flooding).

Political influence was needed to gain the attention and support of FEMA leadership to move forward with recovery activities. Alumni and friends provided financial aid and assistance. Other sources of support for long-term rebuilding efforts included religious organizations, concerts and other benefit events, foundations, and corporate donors. Agencies such as the National Science Foundation (NSF) and National Institutes of Health (NIH) also provided supplements to their grants to move the research forward. Table 1 gives an overview of the multiple partners supporting the financial recovery of Xavier University of Louisiana.

### 4.2. Lessons from Hurricane Harvey—Dong Liang

At Texas Southern University, widespread flooding resulted in the displacement of faculty and staff members from their homes and loss of their vehicles from extensive water damage.

Delays in academic recovery occurred due to disrupted infrastructure and loss of data. Graduate students experienced similar personal losses and family burdens as well as additional challenges, including a two-week delay in the start of classes coupled with associated delays in registration and other administrative activities. Specific infrastructure challenges included an inability to access the university facilities for two weeks, major instrument damage, loss of paper/electronic data, and damage to the animal care facility with subsequent challenges meeting environmental standards.

Nonetheless, the response and recovery were less than what would be anticipated with such extensive flooding. Some proactive mitigating actions included the protection of freezers full of valuable samples using backup generators and the implementation of emergency response plans for the facilities housing research animals using lessons learned from prior events (i.e., Tropical Storm Allison and Hurricane Ike).

Some ongoing help has been required for unanticipated major instrument replacement or repair as well as recalibration. Some building improvements, including flood wall installation, will be needed to mitigate damage from future floods.

### 4.3. Lessons from Hurricanes Irma and Maria in Puerto Rico—Carlos E. Rodríguez-Díaz

The experience of Puerto Rico in the aftermath of these hurricanes can be viewed from the perspective of social determinants of health. Puerto Rico is an archipelago located in the Caribbean, 1000 miles south-east of Florida. Puerto Rico is an organized but unincorporated U.S. territory. As evidenced by a recent poll, nearly half of the adults in mainland U.S. do not know Puerto Ricans are fellow citizens [11], yet Puerto Ricans have been U.S. citizens by birth since 1917. As an unincorporated territory of the U.S., Puerto Rico lacks self-determination, and Puerto Ricans on the islands do not have full representation in Congress and cannot vote for president. Furthermore, due to its territorial status, U.S. federal mandates take precedence over local legislation and policies in all areas of governance.

Certain U.S. policies are relevant to the economic crisis in Puerto Rico. The Jones Act affects shipping of goods to and from the island [12]. In effect, the Jones Act forces Puerto Ricans to pay nearly double the price for U.S. goods through various tariffs, fees, and taxes. The Act mandates goods shipped between U.S. ports to be shipped on American-made and operated ships. These factors affecting the cost of goods received and shipped out of Puerto Rico negatively impact both the base economy and the ability of the island to cover the cost of rebuilding its infrastructure and aiding the currently afflicted residents.

Puerto Rico is still treated as a colony. In the aftermath of Hurricane Maria, issues of governance were evident as was the limited ability to implement emergency preparedness plans [13]. The experience of Puerto Rico is shared with other countries in the region known to be in the “Hurricane Alley”. While certain disasters and public health emergencies can be predicted in this region, it is not only the geographical location that increases the island’s vulnerability to such disasters but also Puerto Rico’s prior colonial status and persistent colonial legacy with its chronic financial and public health implications.

Despite these challenges, within two weeks of the direct impact of Hurricane Maria, the University of Puerto Rico—Medical Sciences Campus (UPR-MSC) was able to resume most of its academic, research, and service activities. This was possible in part due to its privileged location within the Puerto Rico Medical Center. This area was prioritized for re-establishment of services. Moreover, despite personal challenges experienced in the aftermath of the hurricanes, the UPR-MSC personnel and the administration were able to sustain services at a minimal level; however, the global financial situation in Puerto Rico has compromised the overall recovery toward achievement of normal robust operations.

Inadequate systematic health and humanitarian disaster relief following Hurricane Irma and Maria led to outbreaks and exacerbation of infectious diseases [14], limited access to clean water, and malnutrition for many. The implementation of further austerity measures on Puerto Rico’s governmental budget has raised even more concerns about the availability of local resources to address the health care challenges posed by deterioration in the public health infrastructure after the hurricanes. Moreover, the federal response to the emergency in Puerto Rico has been very slow and limited.

If anything has been evidenced in the aftermath of the hurricanes besides the sociopolitical crisis in Puerto Rico, it is the ability of the people of Puerto Rico to overcome adversity. In this process, we have witnessed solidarity and commitment to the restoration of our communities. Academic institutions and health centers and networks are working in solidarity for the restoration of our communities.

This experience must inform how we conduct translational research to address the social determinants of health inequities. Research initiatives and interventions should be aimed not only at the individual but, importantly, at the community and structural levels to improve and sustain healthy environments. Puerto Rico has the capacity to develop and respond with grassroots initiatives that might be more culturally relevant than poorly adapted “evidence-based” interventions developed elsewhere. Academic institutions in Puerto Rico can use existing resource networks and collaborations from other institutions to support capacity building and a research agenda for sustainable public health interventions in Puerto Rico.

### 4.4. Lessons from Hurricanes Irma and Maria in Puerto Rico—Kenira Thompson

The Ponce Health Sciences University made a number of pre-storm preparations, including installation of storm windows, back-up of critical data, physical protection of scientific equipment, removal of items that could be water damaged from the floor, provision of maintenance to backup power generators, full diesel tanks for backup generators, storage of multiple days of food and water for animals in the vivarium, assessment of all patients in clinical trials prior to storm arrival, and advance processing of pending fiscal requisitions, orders, and payroll payments. The post-storm response to Hurricane Maria occurred in several phases.

*Phase 1* represented a survival mode and assessment. Activities included an initial assessment of the facilities; movement of animals from the vivarium to a safe building; coordination of diesel delivery for subsequent indefinite operation of generators; assessment of the status and location of all staff (led by Human Resources, Division Chiefs, Principal Investigators); assessment of the status and location of all students (led by the Students Affairs Office, Deans, Department Director); coordination with local hospitals, airport, Department of Health, and local municipality for needs assessment and capacity; implementation of counseling with mental health services for staff and students; and undertaking an inventory of facility and equipment damages. *Phase 2* was the process of undertaking campus and facility cleanup and sustaining operations, thus resulting in successful continuity of operations. This included coordination with the H.Lee Moffitt Cancer Center in Tampa, FL, a long-time collaborator institution, to transport over 3,500 tumor tissue samples from PR to Tampa, for safekeeping due to power fluctuations on the island. *Phase 3* was Ponce Health Sciences University faculty, staff, students, and other learners’ efforts to engage and lead in local relief efforts and supplemental outreach to the community. Overall, over 6,500 patient visits were completed in the 90 days following the storm, and over 100,000 of supplies were received and distributed by a network of over 300 volunteers. 

Some key lessons learned regarding emergency response equipment and preparation include the following:

- Analog telephone lines are invincible when the digital system is down.
- Non-network photocopiers can help print hardcopy materials.
- A satellite phone and Satellite Internet service can help when local phone services are lost.
- Redundant power sources (e.g., sufficient generator capacity) are critical.
- Confirmation of diesel/gas delivery prior to the event and advanced payment arrangements can support fuel delivery, even if communications go down.
- A list of physical addresses and other contact information for all key personnel expected for an on-campus recovery is critical.
- The stability of the workforce must be maintained, i.e., ice, water, and temporary accommodations must be provided for all.
- Mental health support should be available—stressing that everyone should take one day at a time—every day—hoping for a better tomorrow.

### 4.5. Lessons from Hurricanes Irma and Maria in Puerto Rico—Josefina Romaguera

The impact on the University of Puerto Rico from Hurricanes Irma and Maria has been significant. In the aggregate, buildings were damaged, students and residents were displaced, and already distressed budgets were further strained. At the Cayo Santiago Field Station—home to more than 1000 rhesus monkeys with DNA samples since the 1950s—the infrastructure compromise that occurred may lead to loss of animals and research materials, which are all irreplaceable. Efforts to fortify the facility are needed [15].

There is also a need to train faculty members, residents, and students in terms of local disaster response with respect to preparation, disaster mitigation, and recovery. Areas of focus should include maintaining electricity by using back-up generators and fuel supplies, providing access to water, and ensuring options for transportation. Issues related to specific research challenges include controlling and permitting access of staff to research facilities, ensuring adequate personnel availability, transportation for research participants to continue their clinical trial involvement, and sample collection and transportation to active laboratory facilities. Communication challenges include access to the internet, cell phones, and alternative methods for clinical trial patients to participate in research (e.g., via home visits by research team).

Other thoughts regarding infrastructure preparation include the use of cement buildings rather than wooden structures to mitigate structural damage, providing alternatives for electricity (e.g., solar energy), and maintaining communication diversification options (e.g., analog phones, web-based phones, handheld radios, and ham radios). Specific clinical services that warrant additional consideration include mental health support for anxiety and depression related to loss of life/property and mitigating the risk of mosquito-borne viral infections and disease in the tropical climate (e.g., zika, dengue, chikungunya) and water-borne infectious diseases (e.g., leptospirosis, salmonellosis, typhoid fever). The coordination of public and private resources to support communities and satisfy basic needs is always a challenge, given the competing demands, more limited personnel, and communication challenges.

Two weeks after Hurricane Maria, only 25 (less than 50%) hospitals were operational, only 9.2% of people had electrical power, only 54% had potable water, and only 45% had cell phone service. Thus, redeveloping a functional infrastructure will take considerable time. Helping students re-engage in their studies and scientists re-engage in their research is a challenge and will take time.

### 4.6. Lessons from Hurricanes Irma and Maria in Puerto Rico—Silvia E. Rabionet Sabater

During natural disasters, the elderly are particularly at risk. Nursing homes, community centers, and other resources needed for social interaction and activities of daily living by elders were destroyed or rendered inoperative by Hurricane Maria. The care of elders with chronic diseases, such as cancer, cardiovascular diseases, diabetes, dementia, and stroke, was disrupted and at times became impossible. Access to medicines, preventive and primary care, and acute interventions were also often disrupted.

Efforts to restore health care and support services remains a priority but depends upon migration of the frail and vulnerable population to sites with intact infrastructure that is culturally relevant and fosters community engagement. Clinical research has been compromised by the loss of electricity and technology and due to the pressing urgency of basic care for the vulnerable.

## 5. Conclusions

Lessons learned from prior storm-related disasters are transportable to other vulnerable academic institutions, especially those situated in flood plains and coastal regions. Preparations should be made in advance of storms, both in the short-term (days prior to landfall in the case of hurricanes) and further in advance (months to years in terms of structural remedies and operational contingencies). Although there are some limits to the planning and development of contingencies, academic institutions have shown great resiliency and accommodation to a variety of contingencies that arise during the aftermath of a storm-related disaster. Caring for the internal and external communities associated with each institution will pay long-term dividends in the survival of academic institutions and the communities they serve.

## Figures and Tables

**Table 1:** With A Little Help from Our Friends—Elements of the Xavier University of Louisiana Katrina Flood Financial Recovery.

The broken levy caused by Hurricane Katrina flooded the Xavier University campus as most of its buildings were submerged in eight feet of water. Eighty per cent of its faculty and staff lost their homes. Astonishingly, under the leadership of Dr. Norman Francis, Xavier University was back up and operating five months later, with its faculty and staff living in on-campus trailers. This was possible because of a wide variety of generous supporters who did not want Xavier University to fail, from our youngest supporter, five-year-old Xavier Young—who directed that his birthday gifts be given to the University sharing his name—to the nation of Qatar and its $17.5 million gift. The following examples of assistance may assist the Puerto Rico schools and others in their recovery effort. *Alumni and Friends:* Alumni Clubs from New York to California hosted fundraisers to support the University’s Hurricane Relief Fund. Individual alumni also employed creative ways to generate funds. For example, one alumnus organized a “mini-campaign” with family, fellow church members, fraternity brothers, friends, and neighbors to seek funds. *Schools and Colleges:* Grambling University sent buses to help students and staff evacuate the flooded campus. It also provided office space and equipment so that the displaced Fiscal Services office could get up and operate again. Our Lady of the Lake College in Baton Rouge, the University of Louisiana in Lafayette, and Houston Community College in Texas provided space and other services for displaced staff to set up offices and research labs so they could work while the University was closed. Xavier University of Ohio temporarily hosted the Xavier University of Louisiana website. Many universities across the country allowed our students to attend classes for credit at no cost to the students; research universities hosted our Science, Technology, Engineering and Math (STEM) faculty and gave them labs. Colleges and universities across the country and even elementary and secondary schools solicited funds from their students; African American fraternities and sororities also rallied their members to provide support. *Religious Organizations:* Xavier University of Louisiana is the nation’s only black and Catholic university. As such, the Sisters of the Blessed Sacrament—which founded Xavier University—as well as Diocese and Archdiocese around the country sent contributions. Other religious organizations such as the American Jewish Committee and the United Black Presbyterians and individual churches around the country took up collections and sent funds to Xavier University. *Concerts and Other Benefits:* Organizations organized special concerts and benefits to raise funds. The Office of Black Ministry in the Archdiocese of New York organized its annual Black History Month Celebration into a concert fundraiser for Xavier University of Louisiana featuring gospel vocalist BeBe Winans and enlisting key sponsors such as the National Basketball Players Association. *Foundations, Corporations, and other organizations:* Xavier University of Louisiana also received critical support through grants from organizations such as the Mellon Foundation, the Bush Foundation, the William Randolph Hearst Foundation, the Sherman Fairchild Foundation, the Kresge Foundation, the Charles Stewart Mott Foundation, Pfizer, British Petroleum, the Howard Hughes Institute, and the Wallace Coulter Foundation.
